# Obesity Exacerbates Irritable Bowel Syndrome-Related Sleep and Psychiatric Disorders in Women With Polycystic Ovary Syndrome

**DOI:** 10.3389/fendo.2021.779456

**Published:** 2021-11-16

**Authors:** Ping-Huei Tseng, Han-Mo Chiu, Chia-Hung Tu, Ming-Shiang Wu, Hong-Nerng Ho, Mei-Jou Chen

**Affiliations:** ^1^ Department of Internal Medicine, National Taiwan University Hospital, Taipei, Taiwan; ^2^ College of Medicine, National Taiwan University, Taipei, Taiwan; ^3^ Department of Obstetrics and Gynecology, National Taiwan University Hospital, Taipei, Taiwan; ^4^ Research Center for Cell Therapy and Regeneration Medicine, and College of Medicine, Taipei Medical University, Taipei, Taiwan; ^5^ Livia Shang Yu Wan Chair Professor of Obstetrics and Gynecology, National Taiwan University, Taipei, Taiwan

**Keywords:** polycystic ovary syndrome, irritable bowel syndrome, Rome III, obesity, psychiatric morbidity, sleep disorders

## Abstract

**Background/Objectives:**

Polycystic ovary syndrome (PCOS) and irritable bowel syndrome (IBS) share similar clinical and psychosocial features. We aimed to investigate the clinical characteristics of IBS in women with PCOS, and its relationship with obesity, metabolic and hormonal profiles, as well as sleep and psychiatric disorders.

**Subjects/Methods:**

This is a cross-sectional case-control study of 431 untreated women with PCOS and 259 healthy volunteers. All participants were assessed with a comprehensive clinical evaluation and two questionnaires: the Athens Insomnia Scale (AIS) and the Brief Symptom Rating Scale (BSRS-5). IBS was diagnosed using the Rome III criteria. Obesity was defined as a BMI ≥30 kg/m^2^. Anthropometric measurements, metabolic, hormonal profiles, and psychosocial morbidities were compared.

**Results:**

Women with PCOS were more likely to have IBS (10.7% *vs* 5.8%, *p*=0.029) and obesity (29% *vs* 4%, *p*<0.001) than healthy volunteers. Mixed-type IBS (IBS-M) was the most common subtype (74%) among patients with PCOS and IBS. There was a higher prevalence of psychiatric morbidities (total BSRS-5 score ≥10) in women with PCOS than in healthy women (11.4% *vs* 3.5%, *p*<0.001). Women with PCOS and IBS were more likely to have sleep difficulties (67.4% *vs* 30.9%, *p*<0.001) and psychiatric morbidities (21.7% *vs* 10.1%, *p*=0.019) than those without IBS. Anthropometrics, metabolic and hormonal profiles were similar between PCOS women with and without IBS. Among women with PCOS, those with both IBS and obesity had the highest risk of developing sleep difficulties (odds ratio: 5.91; 95% confidence interval: 1.77–19.77) and psychiatric distress (odds ratio: 4.39; 95% confidence interval: 1.26–15.29) than those without.

**Conclusion:**

Women with PCOS have increased IBS, obesity, sleep and psychiatric disturbances. The presence of IBS in PCOS women is associated with sleep and psychiatric disorders. The coexistence of obesity and IBS exacerbates sleep difficulties and psychiatric distress. Screening and management of IBS and obesity might be warranted to improve sleep and psychiatric disturbances in women with PCOS.

## Introduction

Polycystic ovary syndrome (PCOS) is a common endocrine disorder among women of early reproductive age. It is characterized by chronic anovulation (oligomenorrhea or amenorrhea), clinical or biochemical hyperandrogenism, and the presence of polycystic ovaries (1). Women with PCOS have a higher risk of developing infertility, obesity, insulin resistance, dyslipidemia, hypertension, and metabolic derangement that may lead to subsequent cardiometabolic diseases than women without PCOS (2).

Women with PCOS are also more likely to present with psychological disorders such as depression, anxiety, sexual dysfunction, and psychosocial problems, affecting their health-related quality of life (QOL) (3–5). One study using the Taiwan National Health Insurance Research Database found that the incidence of depressive, anxiety and sleep disorders was higher among patients with PCOS than among those in the comparison cohort (6).

Irritable bowel syndrome (IBS) is characterized by chronic or recurrent abdominal pain or discomfort associated with altered bowel habits in the absence of structural or biochemical abnormalities (7). The prevalence of IBS varies substantially among countries, ethnic populations, and the criteria used to define it. According to the latest meta-analysis, the pooled prevalence of IBS in 53 studies that used the Rome III criteria to diagnose it, from 38 countries and 395 385 participants, is 9.2% (0.4–29.2%) (8). The prevalence of IBS is generally higher in premenopausal women than in men (8). The chronic and relapsing nature of IBS is frequently associated with psychosocial and sleep comorbidities, affecting the patient’s QOL and resulting in frequent hospital visits and increased utilization of healthcare resources (9). Although the pathophysiology of IBS remains unclear, the rapidly increasing incidence in Western countries and Taiwan has been attributed partly to lifestyle changes and increased levels of stress. Even though patients with PCOS and IBS have similar features and risk factors, including young adult women and psychosocial comorbidities, IBS seems to be overlooked in patients with PCOS (10). The related clinical characteristics, such as obesity and metabolic derangement, in this specific population have also been rarely studied with conflicting results (11, 12). Since the respective prevalence rates of PCOS, IBS and obesity are increasing in the Asian population, our primary aim in this study was to investigate the prevalence of IBS, diagnosed using the Rome III criteria, in women with PCOS. In addition, we studied the clinical characteristics, especially the anthropometrics, metabolic and hormonal profiles, of women with PCOS and IBS to clarify the associated risk factors and pathophysiology. Finally, we studied the relationship between IBS, obesity and psychosocial stress and sleep difficulties in women with PCOS.

## Materials and Methods

### Study Design and Participants

We conducted this case-control study at the National Taiwan University Hospital (NTUH), a tertiary medical center. This study was approved by the institutional ethics committee (No. 201907101RINC). All participants provided their written informed consent before they were enrolled in the study. Consecutive women aged older than or equal to 20 years, with a confirmed diagnosis of PCOS and a chief complaint of irregular menstrual cycles, clinical hyperandrogenism, or both, were eligible for enrolment and were approached by the attending physicians in the reproductive endocrinology clinic. The diagnosis of PCOS was made using the Rotterdam criteria, and the enrolment inclusion and exclusion criteria we used for patients with PCOS have previously been described in detail (13). Briefly, the diagnosis of PCOS required at least two of the following three criteria were met: (1) oligomenorrhea (<8 spontaneous menstrual cycles per year at least 3 years before enrollment) or amenorrhea; (2) biochemical hyperandrogenemia (serum total testosterone level.0.77 ng/ml) and (3) polycystic ovaries (.12 follicles [2–9 mm in size] per ovary by transvaginal ultrasonography or an ovarian volume >10 ml per ovary by transabdominal ultrasonography with a distended bladder for virginal women). None of the women had been prescribed medications for their symptoms before enrolment. For the control group, healthy volunteer women who had come to the same institute for a routine health check-up and were older than or equal to 20 years were eligible for enrolment and were invited to participate in this study; the need for healthy volunteers was publicized using advertising messages targeted to the general population. All of the study and control subjects were enrolled after excluding other endocrine, organic and systemic abnormalities, such as hyperprolactinemia, thyroid dysfunction, Cushing’s syndrome, congenital adrenal hyperplasia, an adrenal tumor, an ovarian tumor, autoimmune disease, heart failure, liver cirrhosis, end-stage renal disease, malignancy, central nervous system disease, current or previous use of oral contraceptives within 6 months of enrollment or the use of medications known to affect the hypothalamic–pituitary–ovarian axis (anti-androgens, ovulation induction agents, anti-diabetic medications, anti-obesity medications or glucocorticoids). All healthy volunteers and women with PCOS were screened using a self-administered questionnaire, an in-person interview by a gynecologist, physical examination, and blood biochemical analysis. All participants in the control group had regular ovulatory cycles (mean cycle length 25–32 days) and did not present with any signs of clinical or biochemical hyperandrogenism. Anthropometric data, such as body mass index (BMI) and waist circumference, were measured by certified nurses. For women with PCOS, additional blood samples were taken during the follicular phase to assess their hormonal and metabolic profiles.

### Symptom Evaluation and Diagnosis of IBS

We assessed participants using a validated symptom questionnaire and diagnosed IBS using the Rome III criteria. Diagnosis is based on the presence of recurrent abdominal pain or discomfort, at least 3 days/month, for the last three months with symptom onset for at least six months, combined with at least two of the following conditions: symptoms associated with a change in the frequency of defecation or form of the stool and improvement of pain or discomfort following defecation (14). Participants with IBS were further categorized into four subtypes according to the predominant stool pattern: diarrhea-predominant IBS (IBS-D), constipation-predominant IBS (IBS-C), mixed IBS (IBS-M), and un-subtyped IBS (IBS-U) (14).

### Evaluation of Sleep Difficulties and Psychiatric Distress

All participants completed the validated Chinese version of the Athens Insomnia Scale (AIS) and the Brief Symptom Rating Scale (BSRS-5) to evaluate their sleep difficulties and stress levels. The AIS is a brief self-assessment instrument that consists of eight items: difficulty of sleep induction, awakening during the night, final awakening, total sleep duration, overall sleep quality, well-being, functioning capacity, and sleepiness during the day. It is a validated instrument for diagnosing insomnia (15). The first five items of the AIS measure sleep quantity and quality, and the last three measure daytime dysfunction related to insomnia. If the participants had experienced sleep difficulties at least three times a week during the previous month, they were asked to rate each item from 0 to 3 (0, no problem; 1, minor problem; 2, marked problem; 3, severe problem). The total score for the eight items ranges from 0 to 24. A score ≥ 6 suggests the presence of insomnia.

The BSRS-5 is a five-item screening tool for identifying common psychiatric morbidities such as anxiety, depression, and related disorders (16). Each item’s severity is evaluated using a five-point scale (0, no symptoms; 1, mild; 2, moderate; 3, severe; 4, very severe). The total score for all five items ranges from 0 to 20. A score ≥ 10 indicates moderate to severe psychiatric morbidity that may require psychiatric counselling.

### Statistical Analysis

For the sample size estimation, we planned to enroll 500 women with PCOS and 500 healthy volunteers in a 1:1 ratio based on the earlier pilot study by Mathur et al. (11). Nevertheless, because of the COVID 19 pandemic started since early 2020, the recruitment became much more difficult, especially for the heathy volunteers. The study was early terminated in January 2021. Continuous variables are expressed as mean ± standard deviation (SD) and were compared using Student’s *t*-tests. Categorical variables are expressed as a percentage and were analyzed using chi-squared or Fisher’s exact tests as appropriate. First, we compared the demographic characteristics, anthropometrics, prevalence rates of IBS, and scores for the sleep and mental health questionnaires between women with PCOS and healthy volunteers. Second, to explore the pathophysiology of IBS in PCOS, we compared the anthropometrics and metabolic and hormonal profiles of PCOS patients with and without IBS. Finally, to explore the role of obesity in the association between PCOS, IBS, and psychiatric or sleep disorders, we stratified women with PCOS based on obesity (BMI ≥ 30 kg/m^2^ or < 30 kg/m^2^) and presence of IBS. We compared the groups and used the Mantel–Haenszel test to assess the linear trend. Patients with PCOS but without IBS or obesity were considered the reference group. We calculated the 95% confidence interval (CI) and odds ratios for the other three groups. A *p*-value of < 0.05 was considered statistically significant. All statistical analyses were performed using SPSS 16 (SPSS, Chicago, IL, USA).

## Results

### Participant Characteristics

From September 2019 to January 2021, a total of 431 women with PCOS and 259 healthy women were enrolled in the final analysis. The flow diagram was shown in **Supplementary Figure 1**). Women with PCOS were younger (25.3 ± 4.9 *vs* 48.4 ± 10.5, *p* < 0.001) and had a higher BMI (26.4 ± 6.5 *vs* 23.0 ± 12.6, *p* < 0.001) and larger waist circumference (90.0 ± 14.7 *vs* 77.8 ± 8.5, *p* = 0.024) than women in the control group. There was also a higher prevalence of obesity (29% *vs* 4%, *p* < 0.001) and central obesity (73% *vs* 40%, *p* < 0.001) in women with PCOS than in healthy women (**Table 1**). In the previous three months, the women with PCOS had more abdominal pain or discomfort not related to menstruation (*p* < 0.001) and more hard or lumpy stool (*p* < 0.001) than the control group. In the PCOS group, the overall prevalence of IBS was 10.7%, which was significantly higher than in the control group (5.8%, *p* = 0.029). Mixed-type IBS (IBS-M) was the most common subtype in both groups (**Table 1**).

**Table 1:** Demographic characteristics, anthropometrics, bowel symptom profiles, and prevalence of irritable bowel syndrome in PCOS and control groups.

Characteristics	PCOS	Control	*p*
N	431	259	
Age, y	25.3 ± 4.9	48.4 ± 10.5	<0.001
BMI, kg/m^2^	26.4 ± 6.5	23.0 ± 12.6	<0.001
≥30, n (%)	126 (29)	9 (4)	<0.001
Waist circumference, cm	90.0 ± 14.7	77.8 ± 8.5	0.024
≥80, n (%)	312 (73)	102 (40)	<0.001
**GI symptoms in the last 3 months**			
Abdominal pain or discomfort not related to menstruation, n (%)			<0.001
Never	162 (38)	139 (54)	
Less than one day a month	59 (14)	44 (17)	
One day a month	53 (12)	23 (9)	
Two to three days a month	82 (19)	33 (13)	
One day a week	27 (6)	4 (2)	
More than one day a week	32 (7)	11 (4)	
Every day	16 (4)	5 (2)	
Hard or lumpy stool, n (%)			<0.001
Never or rarely	181 (42)	131 (51)	
Sometimes	189 (44)	79 (31)	
Often	34 (8)	27 (10)	
Most of the time	20 (5)	14 (5)	
Always	7 (2)	8 (3)	
Loose or watery stool, n (%)			0.394
Never or rarely	174 (40)	120 (46)	
Sometimes	205 (48)	110 (43)	
Often	36 (8)	17 (7)	
Most of the time	15 (4)	12 (5)	
Always	1 (0)	0 (0)	
**Diagnosis of IBS (Rome III)**, n (%)	46 (10.7)	15 (5.8)	0.029
Constipation-predominant (IBS-C)	2 (4)	1 (7)	
Diarrhea-predominant (IBS-D)	9 (20)	1(7)	
Mixed (IBS-M)	34 (74)	13 (87)	
Un-subtyped (IBS-U)	1 (2)	0 (0)	

a. Data are presented as mean ± standard deviation or number (percentage).

b. BMI, body mass index; GI, gastrointestinal; IBS, irritable bowel syndrome; PCOS, polycystic ovary syndrome.

c. p < 0.05 indicates statistical significance.

### Comparison of Sleep and Psychological Characteristics Between Patients With PCOS and Healthy Subjects

Women with PCOS had lower scores than the control group for the AIS items ‘awakening during the night’ and ‘early awakening’, but higher scores for ‘sleepiness during the day’ (**Table 2**). These results suggest fewer sleep difficulties during the night but more daytime dysfunction. However, the total AIS scores and the proportion of participants with insomnia (AIS score ≥ 6) did not differ significantly between the groups. The PCOS group had significantly higher scores for three of the BSRS-5 items: ‘feeling easily annoyed or irritated’, ‘feeling depressed’, and ‘feeling inferior to others’ (**Table 2**). Their total scores were also higher (4.46 ± 3.82 *vs* 3.49 ± 3.02, *p* < 0.001), suggesting higher psychosocial stress levels. Additionally, the prevalence of psychiatric morbidity, defined as a total BSRS-5 score of ≥ 10, was higher in the PCOS group (11.4% *vs* 3.5%, *p* < 0.001).

**Table 2:** Sleep and psychological characteristics in the PCOS and control groups, based on the Athens Insomnia Scale (AIS) and the Brief Symptom Rating Scale (BSRS-5).

Characteristics	PCOS	Control	*p*
n	431	259	
**AIS**			
Sleep induction	0.71 ± 0.85	0.68 ± 0.80	0.665
Awakening during the night	0.55 ± 0.69	0.95 ± 0.78	<0.001
Early awakening	0.30 ± 0.60	0.60 ± 0.75	<0.001
Total sleep duration	0.79 ± 0.75	0.86 ± 0.77	0.202
Sleep quality	0.78 ± 0.92	0.88 ± 0.75	0.099
Wellbeing during the day	0.50 ± 0.66	0.49 ± 0.64	0.829
Functioning capacity during the day	0.46 ± 0.67	0.39 ± 0.60	0.177
Sleepiness during the day	0.71 ± 0.70	0.49 ± 0.57	<0.001
Total score	4.78 ± 3.97	5.17 ± 3.93	0.214
Total score ≥ 6, n (%)	150 (34.8)	108 (41.7)	0.070
**BSRS-5**			
Feeling nervous	0.88 ± 0.87	0.80 ± 0.71	0.208
Feeling easily annoyed or irritated	1.04 ± 0.94	0.81 ± 0.76	0.001
Feeling depressed	0.87 ± 0.93	0.65 ± 0.80	0.002
Feeling inferior to others	0.87 ± 0.97	0.40 ± 0.67	<0.001
Sleep difficulties	0.81 ± 1.04	0.89 ± 0.99	0.298
Total score	4.46 ± 3.82	3.49 ± 3.02	<0.001
Total score ≥ 10, n (%)	49 (11.4)	9 (3.5)	<0.001

a. PCOS, polycystic ovary syndrome.

b. p < 0.05 indicates statistical significance.

### Impact of IBS on the Clinical Characteristics of Patients With PCOS

We compared the demographics, metabolic profiles, hormone profiles, and sleep and psychological characteristics of PCOS women with and without IBS (**Table 3**). Women with PCOS and IBS had higher scores for all eight AIS items and higher total scores. Up to 67.4% of patients with PCOS and IBS had insomnia, which was significantly higher than those without IBS (30.9%, *p* < 0.001). Patients with PCOS and IBS also had higher scores for all five BSRS-5 items and higher total scores (**Table 3**). There was a higher prevalence of psychiatric morbidities (21.7% *vs* 10.1%, *p* = 0.019) in patients with PCOS and IBS. Other clinical characteristics (anthropometrics and metabolic and hormonal profiles) were similar in the two groups.

**Table 3:** Anthropometrics, laboratory, and sleep and psychological characteristics in PCOS patients with and without IBS.

Characteristics	PCOS with IBS	PCOS without IBS	*p*
n (%)	46 (10.7)	385 (89.3)	
Age, y	26.3 ± 4.8	25.1 ± 4.9	0.123
BMI, kg/m^2^	26.0 ± 6.7	26.4 ± 6.5	0.701
≥ 30, n (%)	13 (28)	113 (29)	0.878
Waist circumference, cm	89.2 ± 15.4	90.1 ± 14.6	0.704
≥ 80, n (%)	32(70)	280 (73)	0.630
Menstrual cycle interval (days)	154.9 ± 110.6	143.8 ± 111.7	0.524
**Metabolic profile**			
Systolic blood pressure, mmHg	116.7 ± 14.6	116.9 ± 13.9	0.934
Diastolic blood pressure, mmHg	76.0 ± 10.3	75.8 ± 11.5	0.895
Fasting blood glucose, mg/dL	87.1 ± 7.9	85.5 ± 12.1	0.382
Triglycerides, mg/dL	117.3 ± 82.6	100.1 ± 59.3	0.176
Total cholesterol, mg/dL	184.0 ± 30.6	189.3 ± 34.1	0.534
HDL-C, mg/dL	51.5 ± 11.8	54.8 ± 24.6	0.361
LDL-C, mg/dL	106.8 ± 30.4	110.5 ± 31.7	0.443
Uric acid, mg/dL	5.5 ± 1.3	5.4 ± 1.2	0.600
**Hormonal profile**			
FSH (mIU/mL)	6.1 ± 1.6	6.3 ± 2.3	0.611
LH (mIU/mL)	11.0 ± 4.7	11.3 ± 6.3	0.769
E2 (pg/mL)	62.7 ± 40.4	58.2 ± 66.4	0.659
P4 (ng/mL)	0.97 ± 1.93	0.55 ± 1.27	0.155
Testosterone (ng/mL)	0.67 ± 0.35	0.61 ± 0.26	0.242
SHBG (nmol/L)	37.4 ± 25.2	37.0 ± 21.8	0.932
FAI (%)	8.7 ± 5.9	8.3 ± 6.8	0.777
DHEAS (μg/dL)	299.0 ± 108.2	266.5 ± 110.2	0.149
Prolactin (ng/mL)	8.61 ± 3.57	9.51 ± 5.69	0.296
TSH (μIU/mL)	1.76 ± 0.67	1.89 ± 1.07	0.277
**AIS**			
Sleep induction	1.33 ± 1.06	0.63 ± 0.79	<0.001
Awakening during the night	1.02 ± 0.83	0.49 ± 0.65	<0.001
Early awakening	0.57 ± 0.81	0.26 ± 0.57	0.017
Total sleep duration	1.07 ± 0.80	0.75 ± 0.74	0.007
Sleep quality	1.26 ± 0.91	0.72 ± 0.90	<0.001
Wellbeing during the day	0.91 ± 0.92	0.45 ± 0.60	0.002
Functioning capacity during the day	0.87 ± 0.98	0.41 ± 0.61	0.003
Sleepiness during the day	1.11 ± 0.95	0.66 ± 0.65	0.003
Total score	8.13 ± 5.12	4.38 ± 3.61	<0.001
Total score ≥ 6, n (%)	31 (67.4)	119 (30.9)	<0.001
**BSRS-5**			
Feeling nervous	1.35 ± 1.08	0.82 ± 0.82	0.002
Feeling easily annoyed or irritated	1.46 ± 0.96	0.99 ± 0.93	0.001
Feeling depressed	1.24 ± 1.14	0.82 ± 0.90	0.004
Feeling inferior to others	1.24 ± 1.16	0.82 ± 0.84	0.006
Sleep difficulties	1.59 ± 1.29	0.72 ± 0.97	<0.001
Total score	6.87 ± 4.56	4.17 ± 3.62	<0.001
Total score ≥ 10, n (%)	10 (21.7)	39 (10.1)	0.019

a. AIS, Athens Insomnia Scale; BMI, body mass index; BSRS-5, Brief Symptom Rating Scale; DHEAS, dehydroepiandrosterone sulphate; E2, estradiol; FAI, free antigen index, FAI (%) = testosterone (ng/mL) * 3.47 * 100/SHBG (nmol/L); FSH, follicle stimulating hormone; HDL-C, high-density lipoprotein cholesterol; IBS, irritable bowel syndrome; LDL-C, low-density lipoprotein cholesterol; LH, luteinizing hormone; P4, progesterone; PCOS, polycystic ovary syndrome; SHBG, sex hormone binding protein; TSH, thyroid stimulating hormone.

b. p < 0.05 indicates statistical significance.

### Impact of Obesity on the Clinical Characteristics of Patients With PCOS

Because obesity is prevalent in women with PCOS, we compared the clinical characteristics of PCOS women with a BMI ≥ 30 kg/m^2^ and those with a BMI < 30 kg/m^2^ (**Table 4**). The patients with a BMI ≥ 30 kg/m^2^ were more likely to have metabolic and hormonal derangement. Moreover, these women had higher total scores and scores for multiple items for both the AIS and BSRS-5 instruments, suggesting higher levels of sleep and psychiatric distress in this subgroup. Since both IBS and obesity were associated with more sleep and psychological problems in the participants, we stratified the PCOS group based on both IBS and obesity (**Table 5**). Women with PCOS, IBS, and obesity had the highest prevalence of insomnia and psychiatric morbidities (69.2% and 30.8%, respectively), followed by those with IBS only and then those with obesity only. Women without IBS or obesity had the lowest prevalence of insomnia and psychiatric morbidities (27.6% and 9.2%, respectively). Among women with PCOS, those with both IBS and obesity had the highest risk of developing sleep difficulties [odds ratio (OR): 5.91; 95% confidence interval (CI): 1.77–19.77) and psychiatric distress (OR: 4.39; 95% CI: 1.26–15.29) than those without.

**Table 4:** Laboratory, sleep, and psychological characteristics of patients with PCOS stratified according to BMI.

Characteristics	PCOS and BMI ≥ 30 kg/m^2^	PCOS and BMI < 30 kg/m^2^	*p*
n (%)	126 (29.2)	305 (71.0)	
**Metabolic profile**			
Systolic blood pressure, mmHg	127.9 ± 14.8	112.4 ± 10.8	<0.001
Diastolic blood pressure, mmHg	84.2 ± 12.0	72.4 ± 9.2	<0.001
Fasting blood glucose, mg/dL	90.9 ± 18.2	83.5 ± 6.6	<0.001
Triglycerides, mg/dL	142.2 ± 73.1	85.4 ± 48.5	<0.001
Total cholesterol, mg/dL	192.4 ± 38.2	184.7 ± 31.6	0.048
HDL-C, mg/dL	44.9 ± 14.1	58.3 ± 25.5	<0.001
LDL-C, mg/dL	125.6 ± 33.5	103.8 ± 28.4	<0.001
Uric acid, mg/dL	6.4 ± 1.2	5.1 ± 1.0	<0.001
**Hormonal profile**			
FSH (mIU/mL)	6.1 ± 3.2	6.4 ± 1.7	0.320
LH (mIU/mL)	8.0 ± 4.8	12.6 ± 6.1	<0.001
E2 pg/mL)	59.9 ± 49.0	58.2 ± 69.4	0.805
P4 (ng/mL)	0.56 ± 1.11	0.61 ± 1.44	0.702
Testosterone (ng/mL)	0.60 ± 0.27	0.62 ± 0.28	0.478
SHBG (nmol/L)	18.8 ± 7.3	42.1 ± 22.2	<0.001
FAI (%)	13.5 ± 7.8	6.9 ± 5.6	<0.001
DHEAS (μg/dL)	238.0 ± 116.2	279.1 ± 107.2	0.017
Prolactin (ng/mL)	9.61 ± 5.18	9.33 ± 5.64	0.062
TSH (ng/mL)	1.17 ± 1.30	1.75 ± 0.88	<0.001
**AIS**			
Sleep induction	0.81 ± 0.94	0.67 ± 0.80	0.123
Awakening during the night	0.68 ± 0.77	0.50 ± 0.65	0.023
Final awakening	0.31 ± 0.65	0.29 ± 0.58	0.675
Total sleep duration	0.89 ± 0.84	0.75 ± 0.71	0.072
Sleep quality	0.86 ± 0.85	0.75 ± 0.95	0.256
Wellbeing during the day	0.91 ± 0.92	0.45 ± 0.60	0.030
Functioning capacity during the day	0.57 ± 0.72	0.42 ± 0.64	0.041
Sleepiness during the day	0.89 ± 0.73	0.63 ± 0.68	0.001
Total score	5.61 ± 4.34	4.44 ± 3.76	0.005
Total score ≥ 6, n (%)	53 (42.1)	97 (31.8)	0.042
**BSRS-5**			
Feeling nervous	0.97 ± 0.88	0.84 ± 0.86	0.175
Feeling easily annoyed or irritated	1.14 ± 1.01	0.99 ± 0.91	0.152
Feeling depressed	1.09 ± 0.99	0.77 ± 0.89	0.001
Feeling inferior to others	1.06 ± 1.10	0.79 ± 0.91	0.016
Sleep difficulties	0.96 ± 1.09	0.75 ± 1.02	0.056
Total score	5.22 ± 4.20	4.15 ± 3.61	0.013
Total score ≥ 10, n (%)	18 (14.3)	31 (10.2)	0.220

a. AIS, Athens Insomnia Scale; BMI, body mass index; BSRS-5, Brief Symptom Rating Scale; DHEAS, dehydroepiandrosterone sulphate; E2, estradiol; FAI, free antigen index, FAI (%) = testosterone (ng/mL) * 3.47 * 100/SHBG (nmol/L); FSH, follicle stimulating hormone; HDL-C, high-density lipoprotein cholesterol; IBS, irritable bowel syndrome; LDL-C, low-density lipoprotein cholesterol; LH, luteinizing hormone; P4, progesterone; PCOS, polycystic ovary syndrome; SHBG, sex hormone binding protein; TSH, thyroid stimulating hormone.

b. p < 0.05 indicates statistical significance.

**Table 5:** Sleep and psychological characteristics of patients with PCOS stratified according to presence of IBS and obesity (BMI ≥ 30 kg/m^2^).

Characteristics	IBS (−) BMI < 30	IBS (−) BMI ≥ 30	IBS (+) BMI < 30	IBS (+) BMI ≥ 30	*p*
n (%)	272 (63.1)	113 (26.2)	33 (7.7)	13 (3.0)	
**AIS**					
Total score	4.07 ± 3.51	5.12 ± 3.77	7.03 ± 3.82	10.92 ± 6.90	<0.001
Total score ≥ 6, n (%)	75 (27.6)	44 (38.9)	22 (66.7)	9 (69.2)	<0.001 (for trend)
Risk of AIS ≥ 6, OR (95% CI)	1	1.68 (1.06–2.66)	5.20 (2.41–11.24)	5.91 (1.77–19.77)	
**BSRS-5**					
Total score	3.85 ± 3.46	4.93 ± 3.90	6.42 ± 4.04	8.00 ± 5.70	<0.001
Total score ≥ 10, n (%)	25 (9.2)	14 (12.4)	6 (18.2)	4 (30.8)	0.009 (for trend)
Risk of BSRS-5 ≥ 10, OR (95% CI)	1	1.40 (0.70–2.80)	2.18 (0.82–5.78)	4.39 (1.26–15.29)	

a. AIS, Athens Insomnia Scale; BMI, body mass index; BSRS-5, Brief Symptom Rating Scale; CI, confidence interval; IBS, irritable bowel syndrome; OR, odds ratio; PCOS, polycystic ovary syndrome.

b. The Mantel–Haenszel test was used to test the linear trend for statistical significance.

c. p < 0.05 indicates statistical significance.

## Discussion

The main findings of this study are that (1) women with PCOS have a higher prevalence of IBS, as diagnosed using the Rome III criteria, and obesity than healthy controls; (2) women with PCOS and IBS are more likely to have sleep difficulties and psychiatric morbidities; and (3) obesity may increase the impact of IBS to exacerbate sleep and psychiatric disorders in women with PCOS. To our knowledge, this is the first study to report the complex relationship of IBS and obesity in women with PCOS—a population that is increasing continuously—and its relationship with sleep and mental health.

The prevalence of IBS in the control group in this study (5.8%) is consistent with a nationwide questionnaire survey based on the Rome III criteria that was conducted in Taiwan in 2005–2008. The authors of that study reported an overall prevalence of IBS in the general population of 4.4%, and the prevalence was significantly higher in women than in men (5.4% *vs* 3.4%) (17). Our results are also consistent with two previous studies reporting a higher prevalence of IBS in women with PCOS (11, 12). However, the prevalence of IBS in our study is lower, which may be related to the different diagnostic criteria used (Rome I *vs* Rome III) and the populations studied. The Rome I criteria did not categorize IBS into subtypes, but using the Rome III criteria we found that mixed-type IBS was the most common subtype in both groups of our study population. This result is consistent with the latest systematic meta-analysis (8). In contrast, IBS-C was found to be the most common subtype in Iranian women with PCOS (12).

Previous studies have consistently shown that IBS is more common in women than in men (8). The prevalence of IBS subtypes varies according to gender and diagnostic criteria used, however. Women are more likely to present with constipation-predominant IBS (OR: 2.38; 95% CI: 1.45–3.92), according to a meta-analysis based on Rome I or II (18). However, IBS-D was the most common subtype in a study based on the Rome IV criteria.

In the present study, we used the Rome III criteria rather than the more recent Rome IV criteria because they remain the most widely used and well-validated diagnostic criteria in the literature. In addition, the Rome IV criteria include ‘abdominal pain’ and exclude ‘abdominal discomfort’, and also include more stringent frequency criteria. The use of the Rome IV criteria has greatly reduced the prevalence of reported IBS, so this system may be less suitable for an epidemiological survey such as this one (8, 19).

Although the exact pathophysiology of IBS remains elusive, previous studies have suggested that sex hormones and gender differences may play pivotal roles in its development (20). Sex hormones may affect peripheral and central regulatory mechanisms involved in the pathophysiology of IBS and thus alter visceral perception and motility, intestinal barrier function, and immune activation of the intestinal mucosa. Sex hormones also directly affect the gut microbiota and the enteric nervous system (20, 21). Menon et al. have shown that the status of estrogen receptor β affects the composition of the gut microbiota of female mice and that these microbiota respond differently to changes in diet complexity (22). Another study utilizing a non-obese diabetic mouse model also shows that the enhancement of testosterone production in pubescent male mice may induce changes in the gut microbiota, which consequently protects against autoimmune disease (23). Several animal studies have shown that estrogens exert a peripheral effect on smooth muscle contractility *via* the inhibition of RhoA signaling, cholecystokinin (CCK) and CCK(A) receptor activation (24).

Bowel dysmotility has also been suggested to be an important contributing factor in the development of IBS. Clinical observations have found that women tend to have slower gastrointestinal (GI) transit and are more prone to constipation than men. However, previous studies have obtained contradictory findings regarding the effect of the various sex hormones on GI motility (25–27). A recent study based on female dihydrotestosterone (DHT)-treated PCOS rats found lower maximal colon muscle contractility in response to acetylcholine stimulation in DHT-treated rats than in untreated rats (28). This lower maximal muscle contractility was found to be associated with extracellular calcium levels. This effect occurs partly *via* a reduction of the responsiveness of acetylcholine and through phosphorylation of 20-kDa regulatory myosin light chain (MLC20) (28). These findings suggest that hyperandrogenism might be involved in bowel dysmotility, resulting in IBS in women with PCOS. However, we did not find any significant differences between the levels of various hormones in PCOS women with or without IBS. It is possible that different androgens and their metabolites have different levels of potency, which may explain these results. It is also possible that factors other than the hormones we assayed may contribute to the higher prevalence of IBS in women with PCOS, and this warrants further investigation.

Several studies have highlighted the association between the abundance of gut microbiota and PCOS in humans (29–31). Another study showed that the diversity and composition of gut microbiota in young adults are affected by the combination of sex, sex hormone concentrations, and obesity, and that this has specific consequences in women with PCOS (32). It has also been suggested that the gut microbiota play a key role in modulating the gut–brain interaction and intestinal barrier functioning and, therefore, that they may participate in the pathogenesis of IBS (33). Alteration of the gut microbiome could lead to low-grade inflammation and immune dysfunction in the intestinal mucosa, resulting in changes in intestinal secretion, visceral perception, and intestinal dysmotility in patients with IBS (34). In addition, small intestinal bacterial overgrowth (SIBO), a clinical syndrome featuring an abnormally high number or abnormal type of bacteria in the small intestine, has been associated with the pathophysiology of IBS, and it causes abdominal pain, bloating, diarrhea, steatorrhea, and flatulence (35). Most studies and recent meta-analyses have reported a higher frequency of SIBO among patients with IBS than in controls (36, 37). Therefore, we speculate that gut microbiota dysbiosis in patients with PCOS also contributes to the higher prevalence of IBS in these patients. Further studies to compare the composition of gut microbiota between PCOS women with and without IBS may help to clarify this relationship.

Psychosocial distress is common in both patients with IBS and patients with PCOS, and could also play a bidirectional role in the development of IBS in women with PCOS (6). A recent meta-analysis reported that patients with IBS have more frequent and severe depressive symptoms than healthy controls (38). Further meta-analysis regressions reveal that younger women (the group at most risk for PCOS) have significantly more severe depressive symptoms (38). The close association between psychiatric disorders and IBS has been explained by the ‘gut–brain axis’ hypothesis. Chronic psychological stress has been associated with GI dysmotility, increased intestinal permeability, and visceral hyperalgesia in animal and human studies (39–41). One study demonstrated that corticosterone mediates the chronic psychological-stress-induced increase in intestinal permeability in rats by decreasing the expression of tight junction proteins (39). Depressive symptoms were also found to affect pain thresholds in the alternating IBS subtype (42). Studies based on a nationwide Swedish cohort and hospital admissions found that women with PCOS have an increased risk of depression and anxiety disorders (43, 44). A meta-analysis of 30 cross-sectional studies, representing 3050 patients with PCOS and 3858 controls from 10 different countries, demonstrated a significantly increased risk of moderate and severe depressive and anxiety symptoms in women with PCOS (5). Therefore, PCOS patients are likely to suffer from additional psychiatric distress when they also suffer from troublesome bowel symptoms. In the present study, women with PCOS had a significantly higher prevalence of psychiatric morbidities than healthy volunteers. In addition, women with PCOS and IBS had higher psychiatric distress than patients with PCOS but not IBS. Up to 21.7% of women with PCOS and IBS had moderate psychiatric morbidities. Since IBS symptoms may be relieved by lifestyle modification and pharmacological treatment, further research is required to investigate whether screening and treatment of IBS in women with PCOS may provide psychiatric relief.

Obesity is an important feature of PCOS. Previous studies have found that women with PCOS and IBS had a higher BMI (32.9 ± 2.0 kg/m^2^) than those with PCOS but no IBS (30.3 ± 1.6 kg/m^2^) (11). Although our participants with PCOS had a higher BMI than healthy volunteers, the BMI of PCOS women with and without IBS was similar. The BMI of our participants was somewhat lower (26.4 ± 6.5 kg/m^2^) than in Western studies, suggesting that obesity alone may not play a significant role in the development of IBS in women with PCOS. However, obesity has been associated with increased psychiatric disorders and sleep disturbances in previous studies (45, 46). In one of the aforementioned meta-analyses, women with PCOS and concurrent psychiatric disorders such as depression or anxiety had a higher BMI, suggesting that obesity plays a role in the association between PCOS and psychiatric disorders (5). Similarly, in a community-based sample population, sleep disturbances were almost twice as common in women with PCOS than in women of similar age without PCOS (47). This association was accounted for by body weight and depressive symptoms. This supports our present findings that the women with PCOS and both obesity and IBS had the most severe sleep problems and psychosocial distress.

This study’s strengths include a relatively large sample of women with PCOS and a complete clinical assessment, including anthropometric measurements and hormonal assays, which allowed for a comprehensive subgroup analysis. In addition, we recruited the control group from the general population who attended the routine health check-up. The in-person interviews and physical examination by gynecologist as well as detailed laboratory and imaging studies to exclude any organic lesions which may interfere the manifestations of IBS symptoms and psychosocial comorbidities. However, our study also has several limitations. First, it was designed as a cross-sectional case-control study, and as such it cannot explain the cause–effect relationship between PCOS and IBS. Second, women with PCOS were younger and more obese than the control group, and age and BMI may be confounding factors in interpreting the higher prevalence of IBS in women with PCOS. However, the fact that our study had mostly younger women reflects the real-world situation, and further this aspect is consistent with two similar studies (11, 12). Future studies with age- and BMI-matched controls may help to clarify this important issue. Finally, our study was conducted in an ethnically Chinese population, and it may not be generalizable to other ethnic populations. Further studies to confirm our findings are thus warranted.

## Conclusions

In conclusion, our study shows that women with PCOS have increased IBS, obesity, sleep and psychiatric disturbances. The presence of IBS in women with PCOS is highly associated with presence of sleep and psychiatric disorders. The coexistence of obesity and IBS exacerbates sleep difficulties and psychiatric distress in women with PCOS. Screening and management of IBS and overweight/obesity might be warranted to improve sleep and psychiatric disturbances in women with PCOS.

## Data Availability Statement

The raw data supporting the conclusions of this article will be made available by the authors, without undue reservation.

## Ethics Statement

The studies involving human participants were reviewed and approved by National Taiwan University Hospital (NTUH) Research Ethics Committee (REC). The patients/participants provided their written informed consent to participate in this study.

## Author Contributions

P-HT, H-MC, C-HT, and M-JC: drafting the manuscript. P-HT, H-MC, and M-JC: study concept and design. P-HT, H-MC, and M-JC: acquisition of data, analysis, and interpretation of data. M-SW, H-NH, and M-JC: critical revision of the manuscript for important intellectual content. P-HT and M-JC: statistical analysis. P-HT, M-SW, and M-JC: obtained funding. M-SW, H-NH, and M-JC: study supervision. All authors have seen and approved the submission of this version of the manuscript and take full responsibility for the manuscript. All authors have given final approval of the version submitted, and agree to be accountable for all aspects of the work in ensuring that questions related to the accuracy or integrity of any part of the work are appropriately investigated and resolved.

## Funding

This study was supported by research grants from the National Taiwan University Hospital (NTUH. 106-003411) and the Ministry of Science and Technology, Taiwan (MOST 109-2314-B002-125-MY3, MOST 105-2325-B-002-041- and MOST 107-2314-B-002-050-). The funders had no role in the study design, data collection and analysis, decision to publish, or manuscript preparation.

## Conflict of Interest

The authors declare that the research was conducted in the absence of any commercial or financial relationships that could be construed as a potential conflict of interest.

## Publisher’s Note

All claims expressed in this article are solely those of the authors and do not necessarily represent those of their affiliated organizations, or those of the publisher, the editors and the reviewers. Any product that may be evaluated in this article, or claim that may be made by its manufacturer, is not guaranteed or endorsed by the publisher.

## Supplementary Material

Supplementary Figure 1 Flow diagram of this study.
